# Comment on "The relantionship of FricTest responses with an urticaria activity score, urticaria control test and quality of life scales in patients with symptomatic dermographism" - Reply^؆^

**DOI:** 10.1016/j.abd.2026.501288

**Published:** 2026-01-30

**Authors:** Mustafa Tosun, Ömer Faruk Kıraç, Rukiye Yasak Güner, Melih Akyol

**Affiliations:** a Department of Dermatology, Faculty of Medicine, Sivas Cumhuriyet University, Sivas, Turkey; b Department of Dermatology, Osmaniye Düziçi Hospital, Osmaniye, Turkey

Dear Editor,

We sincerely thank Dr. Prajnasini Satapathy and colleagues for their interest in our article and for their constructive observations. We appreciate the opportunity to clarify certain methodological aspects and the clinical interpretation of our findings.

Regarding the statistical approach, our study used the Spearman correlation test, as the Kolmogorov-Smirnov analysis indicated a non-parametric data distribution. Although confidence intervals for correlation coefficients were not provided, both the p-values and correlation magnitudes were transparently reported to indicate the direction and strength of the associations. In future studies with larger and more diverse populations, we intend to include 95% Confidence Intervals to improve precision and reproducibility.

The purpose of our study was exploratory in nature ‒ to examine whether FricTest® responses correlate with validated disease activity and quality-of-life instruments such as UAS, UCT, and DLQI. We fully agree that regression modeling would offer additional insight into predictive value; however, given the limited sample size (n = 71) and single-center design, such analyses would have risked model overfitting. Future multicenter studies with larger cohorts are planned to evaluate whether FricTest® thresholds independently predict treatment response after controlling for clinical confounders.

The lack of correlation between FricTest® changes and VAS scores can be explained by the different domains these measures assess. FricTest® quantifies objective mechanical wheal reactivity, whereas VAS reflects patients’ subjective symptom perception, such as itch or burning. Similar discrepancies between objective and subjective parameters have been reported in other studies of chronic inducible urticaria.^1^, ^2^ This divergence highlights that FricTest® primarily captures a physical threshold of dermographism rather than subjective disease burden.

We also emphasize that our study did not aim to demonstrate the superiority of FricTest® over established tools, but rather to explore its complementary role. The finding that 4 mm and 4.5 mm tips correlated best with changes in UCT and DLQI supports the clinical utility of FricTest® as an adjunctive monitoring instrument, potentially refining objective assessment in symptomatic dermographism.

As acknowledged in the article, the limited sample size and single-center setting constrain generalizability. Nevertheless, this pilot study was conducted within a certified UCARE center to generate preliminary data for larger validation projects.

In conclusion, we are grateful for the readers’ valuable comments. We share the view that objective and standardized measures such as FricTest® may enhance disease activity monitoring when interpreted alongside patient-reported outcomes. We hope our response clarifies the study’s intent and methodological framework and contributes to an informed discussion on quantitative tools in chronic inducible urticaria.

## ORCID ID

Mustafa Tosun: 0000-0002-6189-8016

Ömer Faruk Kıraç: 0009-0000-2721-6010

Rukiye Yasak Güner: 0000-0002-5154-4652

Melih Akyol: 0000-0001-7912-0651

## Financial support

None declared.

## Authors' contributions

Ömer Faruk Kıraç: Writing, methodology, data curation, software, contributed to the clinical follow-up and management of patients.

Mustafa Tosun: Formal analysis, writing, approval of final manuscript, validation, and visualization.

Rukiye Yasak Güner: Writing, methodology.

Melih Akyol: Formal analysis, approval of the final manuscript.

## Research data availability

Does not apply.

## Conflicts of interest

None declared.

## References

[1] Mlynek A., Vieira dos Santos R., Ardelean E., Weller K., Magerl M., Church M.K., Maurer M. (2013). A novel, simple, validated, and reproducible instrument for assessing provocation threshold levels in patients with symptomatic dermographism. Clin Exp Dermatol..

[2] Can P.K., Etikan P., Kızıltaç U., Kızıltaç K., Singer R., Kocaturk E. (2019). Fric test revisited: a suggestion for a new scoring system and ıts correlation with urticaria control test and dermatology life quality ındex. Int Arch Allergy Immunol..

